# Escore de Risco Clínico Simples para Prever a Mortalidade Pós-Alta Hospitalar em Pacientes Chineses Hospitalizados por Insuficiência Cardíaca

**DOI:** 10.36660/abc.20200435

**Published:** 2021-08-02

**Authors:** Lei Wang, Li-Qin Wang, Mo-Li Gu, Liang Li, Chen Wang, Yun-Feng Xia

**Affiliations:** 1 Departamento de Medicina Geriátrica Fourth Medical Center Chinese PLA General Hospital Beijng China Departamento de Medicina Geriátrica, the Fourth Medical Center, Chinese PLA General Hospital, Beijng - China; 2 Departamento de Enfermagem Eighth Medical Center Chinese PLA General Hospital Beijng China Departamento de Enfermagem, the Eighth Medical Center, Chinese PLA General Hospital, Beijng - China

**Keywords:** Insuficiência Cardíaca, Pontuação de Propensão, Mortalidade, Alta Hospitalar, Epidemiologia

## Abstract

**Fundamento:**

Doenças cardiovasculares são a principal causa de morte na China. Entretanto, os esforços atuais para se identificar os fatores de risco de morte em pacientes hospitalizados com insuficiência cardíaca (IC) estão direcionados principalmente para a mortalidade durante a internação e a mortalidade após 30 dias nos Estados Unidos. Dessa forma, é necessário um modelo semelhante ao modelo utilizado para prever o risco considerado para procedimentos cirúrgicos cardiovasculares em pacientes para avaliar o risco de pacientes internados com diagnóstico de IC.

**Objetivo:**

Identificar variáveis que podem prever a mortalidade por IC um ano após a alta hospitalar, e desenvolver um escore de risco para avaliar o risco de morte no período de um ano.

**Métodos:**

No presente estudo, 1.742 pacientes chineses com IC foram divididos aleatoriamente em dois grupos: um grupo de amostra de derivação e um grupo de amostra de teste. O método de simulação Monte Carlo via Cadeias de Markov foi usado para identificar variáveis que podem prever a mortalidade um ano após a alta hospitalar. Variáveis com uma frequência >1% na análise bivariada, e que foram consideradas clinicamente significativas, foram qualificadas para análises de modelagens posteriores. A probabilidade posterior de que uma variável estava estatística e significativamente associada ao resultado foi calculada como o número total de vezes em que o IC de 95% da variável não coincidiu com 1 (ou seja, o ponto de referência), dividido pelo número total de iterações. Uma variável com uma probabilidade de 0,9 ou mais alta foi considerado um fator de risco robusto para prever o resultado, e foi incluída na lista final de variáveis. O nível de significância estatística adotado foi 5%.

**Resultados:**

Cinco variáveis que pudessem prever de maneira robusta a mortalidade um ano após a alta hospitalar foram identificadas: idade, sexo feminino, escore da *New York Heart Association* (Associação de Cardiologia de Nova Iorque) >3, diâmetro do átrio esquerdo, e índice de massa corporal. Os modelos de derivação e de teste tiveram uma área de curva característica de operação do receptor de 0,79. Essas variáveis selecionadas foram utilizadas para avaliar o escore de risco de mortalidade por IC após um ano, e este foi dividido em três grupos (baixo, moderado e alto). O grupo de alto risco corresponde a aproximadamente 86% das mortes, e o grupo de risco moderado corresponde a 12% das mortes.

**Conclusão:**

Um escore de risco de 5 variáveis simples pode ser utilizado para avaliar a mortalidade um ano após a alta hospitalar de pacientes internados com IC.

## Introdução

Doenças cardiovasculares são a principal causa de morte na China, correspondendo a aproximadamente 22,5% de todas as mortes.^1^ A insuficiência cardíaca (IC) é a décima-segunda principal causa de internação hospitalar na China, e quatro milhões de chineses sofrem dessa doença.^1^ No geral, a IC na China tem um prognóstico especialmente ruim, com até 40% dos pacientes morrendo por IC no período de um ano.^1^ A carga financeira da IC também é considerável.^2^

Entretanto, os esforços atuais para se identificar os fatores de risco de morte em pacientes hospitalizados por IC, tais como o escore de risco de Framingham, estão voltados principalmente para a mortalidade durante a internação hospitalar^3 , 4^ e a mortalidade após 30 dias nos Estados Unidos.^5 , 6^ Como a IC é uma doença crônica, a identificação dos fatores de risco de mortalidade de pacientes com IC no longo prazo poderia beneficiar os pacientes. Um modelo semelhante ao modelo utilizado para prever o risco considerado para procedimentos cirúrgicos cardiovasculares em pacientes pode ser utilizado para avaliar o risco de pacientes internados com diagnóstico de IC.^7^ Considerando-se o aumento da carga de IC na China, é importante encontrar meios para a estratificação de pacientes com base no risco, no momento do diagnóstico inicial e no momento da alta. Além disso, como a população asiática representa aproximadamente 5% da população dos Estados Unidos e a população chinesa representa 20% da população mundial, os escores de risco desenvolvidos com base em populações ocidentais geralmente estimam incorretamente o risco para populações asiáticas.^8^ Portanto, é importante desenvolver ferramentas clinicamente relevantes para chineses e outros grupos asiáticos. Uma ferramenta que pudesse especificamente indicar a probabilidade de mortalidade após um ano em pacientes chineses com IC teria grande utilidade clínica, pois teria o potencial de orientar a tomada de decisão clínica e de identificar pacientes que têm mais probabilidade de precisar de um monitoramento pós-alta intenso. Além disso, como a raça, a etnia, e o país de origem têm um grande impacto nos resultados clínicos, é importante desenvolver uma avaliação de risco específica para o grupo de interesse, ou seja, os pacientes chineses com IC.

O presente estudo tem o objetivo de identificar fatores de risco que estão mais fortemente correlacionados à mortalidade após um ano entre pacientes chineses com IC, e de desenvolver um escore de risco simples para avaliar o risco de mortalidade um ano após a alta hospitalar nesses pacientes.

## Métodos

### Sujeitos

O presente estudo foi aprovado pelo Comitê de Ética de nosso hospital, e todos os pacientes assinaram o termo de consentimento informado.

O coorte do estudo foi extraído do *Beijing Monitoring Heart Failure Patients and Building Heart Failure Management Network Study* (Estudo de monitoramento de pacientes com insuficiência cardíaca e de construção de rede de gestão da insuficiência cardíaca de Pequim), que incluiu todos os pacientes com idade ≥20 anos hospitalizados por IC em dos 14 hospitais designados em Pequim, na China, entre 10 de outubro de 2015 e 9 de outubro de 2017. Esses pacientes foram divididos aleatoriamente em dois grupos usando um método de tabela aleatória: um grupo de amostra de derivação e um grupo de amostra de teste. As informações sobre mortalidade após um ano foram obtidas por entrevistas telefônicas pós-alta hospitalar.

### Candidatas a variáveis de risco e variáveis de resultado

As candidatas a variáveis de risco incluíram as características demográficas (idade, sexo, e índice de massa corporal [IMC]), histórico médico e comorbidades, fatores de estilo de vida, cirurgias cardíacas prévias, achados clínicos e resultados de testes laboratoriais. A idade e o IMC foram medidos como variáveis contínuas, enquanto sexo foi codificado como feminino (sim/não). O histórico médico incluiu histórico de infarto do miocárdio agudo (IMA), histórico de IC, e histórico de doença cardíaca coronária (DCC), diabetes tipo I ou tipo II, e hipertensão. Cirurgias cardíacas prévias incluíram cirurgias de válvula anteriores. Os achados clínicos incluíram o escore de classificação funcional da *New York Heart Association* (NYHA) (classe >3) e fração de ejeção ventricular esquerda (<40%). Fatores de estilo de vida incluíram histórico de tabagismo, tabagismo atual e consumo de álcool. Os dados de testes laboratoriais incluíram a frequência cardíaca, a pressão arterial, e o diâmetro do átrio esquerdo (em milímetros), que foi medido como variável contínua.

A variável de resultado foi a mortalidade por IC um ano após a alta hospitalar, definida como morte por qualquer causa que tenha ocorrido após uma internação hospitalar por IC registrada. As informações sobre mortalidade foram obtidas por meio de entrevistas telefônicas com esses pacientes. A última entrevista foi realizada em 19 de fevereiro de 2019. Se um paciente tivesse morrido dentro de um ano após a alta, a data da morte era obtida de familiares. Pacientes foram excluídos da amostra do estudo quando nem eles, nem seus familiares puderam ser contactados.

### Análise estatística

Os dados do grupo de amostra de derivação e o grupo de amostra de teste foram comparados pelo teste qui-quadrado em relação às variáveis categóricas e testes t não pareados em relação às variáveis contínuas. Em seguida, o julgamento clínico e a análise de correlação bivariada de Spearman foram utilizados para identificar candidatas a variáveis que possam estar associadas à mortalidade um ano após a alta hospitalar. Variáveis com uma frequência >1% na análise bivariada, e que foram consideradas clinicamente significativas, foram qualificadas para análises de modelagens posteriores. Para as observações em que faltavam dados, foi criada uma variável dummy para atribuir um valor de 0 quando o valor da variável estava presente, e um valor de 1 quando a variável não estava presente. Em seguida, os valores que faltavam foram substituídos pela mediana dos valores presentes daquela variável contínua, e as variáveis contínuas e dummy foram incluídas no modelo. Esse método para modelagem dos dados faltantes considerou que esses dados faltavam aleatoriamente, e permitiu a inclusão de todos os casos disponíveis, embora não tenha sido tão eficiente quanto procedimentos de imputação múltipla.

O método de simulação Monte Carlo via Cadeias de Markov (MCMC) foi usado juntamente com a técnica de regressão logística para identificar um conjunto final de fatores de risco que podem prever a mortalidade por IC um ano após a alta hospitalar. As simulações foram feitas com 10.000 iterações para a amostra de derivação, e um modelo logístico foi ajustado a cada iteração, produzindo um conjunto de variáveis que são “estatisticamente significativas”, ou associadas ao resultado. Dessa forma, 10.000 iterações da simulação produziram 10.000 conjuntos de razões de chance (RC) e intervalos de confiança (IC) de 95%, indicando o nível de significância da associação de cada variável com o resultado. A probabilidade posterior de que uma variável estava estatisticamente e significativamente associada ao resultado foi calculada como o número total de vezes em que o IC de 95% da variável não coincidiu com 1 (ou seja, o ponto de referência), dividido pelo número total de iterações. Uma variável com uma probabilidade de 0,9 ou mais alto foi considerado um fator de risco robusto para prever o resultado, e foi incluído na lista final de variáveis. Esse método foi utilizado em outros estudos.^9^ A área sob a curva característica de operação do receptor (ROC) foi calculada para cada modelo ajustado por iteração, para se avaliar seu poder discriminatório.^10^

### Desenvolvimento do escore de risco

Com base nos resultados da simulação, foi construído um escore de risco simples, baseado em variáveis selecionadas para avaliar a mortalidade após um ano. Cada variável foi ponderada utilizando-se o coeficiente padronizado (CP) específico da variável obtido de um modelo logístico baseado na amostra de derivação original, com a mortalidade após um ano como resultado, e as variáveis selecionadas como variáveis independentes. O CP, que mediu a mudança de coeficiente para uma alteração de desvio padrão (DP) na variável independente, teve o objetivo de representar a importância relativa da variável independente num modelo de regressão. Isso permite fazer a comparação entre variáveis independentes utilizando-se unidades comuns. O escore de risco para cada observação, nas amostras de derivação e de validação, foi calculado como: Escore = ∑ Peso_i_ ⋅ Variável_i_, em que peso_i_ = SC_i_ / ∑ |SC|, e i = 1, 2, 3, …, número total de variáveis selecionadas finais. Em seguida, cada peso foi redimensionado por 100 para permitir que o escore seja uma prática de fácil utilização, com exceção do peso da idade, que é dimensionado apenas em 10. Cada peso foi posteriormente arredondado para cima ou para baixo, até seu número inteiro mais próximo em intervalos de 5 (por exemplo, 32,5 para 30,0, ou 18,0 para 20,0). A idade foi arredondada para baixo até seu patamar mais próximo, com um ponto decimal. Por último, uma base de 100 foi adicionada ao risco para garantir que não houvesse valores negativos nos escores. Para validar e testar esse escore de risco, dois modelos logísticos foram ajustados. Um modelo utilizou variáveis individuais que foram selecionadas na simulação MCMC como variáveis independentes, e o outro modelo utilizou o escore de risco como variável independente. Esses dois modelos foram ajustados com amostras de derivação e de teste, e os valores de r quadrado e ROC foram calculados de ambos os modelos para avaliar o desempenho do escore de risco. Todos os testes estatísticos foram bilaterais e tiveram um nível de significância de 5%, e todas as análises foram realizadas utilizando-se a o software SAS versão 9.3 versão de 64 bits (SAS Institute Inc., Cary, Carolina do Norte, EUA). Todas as variáveis contínuas foram distribuídas normalmente como testadas pelos testes de Shapiro-Wilk. As variáveis contínuas com distribuição normal são descritas utilizando-se média e desvio padrão.

## Resultados

### Características do paciente

O coorte final do estudo incluiu um total de 1.742 pacientes com IC. Entre esses pacientes, as amostras de derivação e de teste incluíram 882 e 860 pacientes, respectivamente. A idade média (DP) do coorte foi de 57,0 (12,5) anos, e 9,5% desses pacientes tinham 40 anos de idade ou menos, enquanto 30,9% desses pacientes tinham idade igual ou superior a 65 anos. Além disso, 19,9% desses pacientes eram mulheres. As características dos pacientes nas amostras de derivação e de validação eram comparáveis ( Tabela 1 ). Não houve diferenças significativas entre esses dois grupos em termos de idade, sexo, diâmetro do átrio esquerdo, frequência cardíaca, FEVE <40 (%), IMA (%), NYHA >3 (%), DCC (%), DM (%), HTN (%), cirurgia de válvula (%), histórico de tabagismo (%), consumo de álcool (%), e mortalidade após um ano (%). Entretanto, não houve diferenças significativas entre esses dois grupos em termos de frequência cardíaca >100 (%) e IC (%). Não houve diferenças significativas em termos de medicamentos e consultas médicas durante o acompanhamento, nem diferenças significativas em resultados de testes laboratoriais entre todos os pacientes.

**Tabela 1:** – Características de linha de base da população do estudo

Características do paciente	Geral (n=1742)	Amostra de derivação (n=882)	Amostra de teste (n=860)	Valor de p
Dados demográficos Idade, média (DP) anos	57,1 (12,4)	57,0 (12,4)	57,1 (12,5)	0,9295
Idade faltante, n=4 (%)	0,2	0,2	0,2	0,9798
Feminino (%)	19,9	19,8	19,9	0,9823
IMC, média (DP)	25,1 (3,4)	25,1 (3,4)	25,0 (3,4)	0,3455
IMC faltante, n=0 (%)	0	0	0	
**Achados clínicos e testes laboratoriais**				
Diâmetro do átrio esquerdo (mm), média (DP)	39,9 (7,9)	40,0 (8,1)	39,9 (7,7)	0,823
Tamanho do átrio esquerdo faltante, n=66 (%)	3,8	4,2	3,1	0,1611
Frequência cardíaca, média (DP)	73,0 (14,0)	73,3 (14,8)	72,7 (13,2)	0,8762
Frequência cardíaca faltante, n=12 (%)	0,7	0,5	0,9	0,2291
Frequência cardíaca >100 (%)	4,5	5,6	3,4	0,0276
FEVE <40 (%)	35,5	35,8	35,1	0,7564
FEVE faltante, n=0(%)	0	0	0	
**Histórico médico**				
IC (%)	18,1	20,2	15,8	0,0177
IMA (%)	22,6	23,4	21,9	0,4557
NYHA >3(%)	42,7	42,6	42,8	0,9461
DCC (%)	71,2	70,4	72,0	0,4699
DM (%)	27,8	27,2	28,4	0,5885
TxC (%)	63,6	62,7	64,5	0,4258
Cirurgia de válvula (%)	6,1	6,8	5,4	0,2044
**Estilo de vida**				
Tabagismo (%)	57,8	57,6	57,9	0,8956
Consumo de álcool (%)	35,1	34,5	35,8	0,5561
Mortalidade após um ano (%)	6,5	7,3	5,8	0,2236

*NYHA: Escore de classificação funcional da New York Heart Association, IMC: Índice de massa corporal, DCC: Doença cardíaca coronária, IC: Insuficiência cardíaca, IMA: Infarto do miocárdio agudo, DM: Diabetes tipo I ou tipo II, HTN: Hipertensão. Dados contínuos foram analisados por teste t. Dados categóricos foram analisados por teste qui-quadrado. P<0,05 é significativo.*

### Variáveis de risco para previsão da mortalidade por IC após um ano

**Figura 1:**
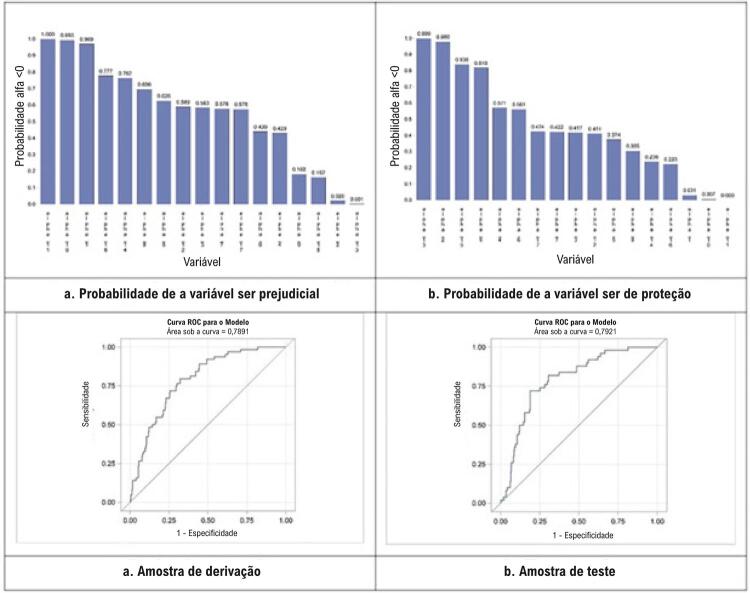
– *Seleção variável e desenvolvimento de modelo para escore de risco.*

Os índices de mortalidade após um ano observados nas amostras de derivação e validação foram 7,3% e 5,8%, respectivamente (p = 0,2236). A Figura 1 apresenta a probabilidade de que cada variável seja associada à mortalidade por IC após um ano. Cinco variáveis, incluindo idade, sexo feminino, IMC, diâmetro do átrio esquerdo, e classe NYHA >3, tinham uma probabilidade de 0,9 ou mais alta de associação significativa com a mortalidade após um ano. Elas foram identificadas como as variáveis finais ( Figuras 1a e 1b ). A Tabela 2 ilustra as RC, CP, e IC de 95% para cada uma das cinco variáveis selecionadas no conjunto de dados de derivação. A área sob a curva ROC desse modelo baseado em 5 variáveis foi 0,789, com um r quadrado de 0,1761 e uma bondade do ajuste de 0,9013. A capacidade preditiva variou de 0,04, no decil mais baixo, a 0,43 no decil mais alto, indicando que o modelo tem bom poder discriminatório ( Figura 1c ). Esse modelo também se comportou de maneira semelhante ao conjunto de dados de teste ( Figura 1d e Tabela 3 ).

**Tabela 2:** – Fatores de risco e pesos correspondentes no escore de risco clínico

Características do paciente	Probabilidade posterior*	Razões de chance (IC 95%)	Coeficiente padronizado	Peso inicial	Direção	Peso redimensionado
Idade, anos	0,969	1,03 (1.01-1.05)	0,1864	0,155891946		1,5
Feminino	0,02	0,44 (0.20-0.97)	-0,1796	0,150204901	(-)	-15
diâmetro do átrio esquerdo (mm)	0,993	1,05 (1.02-1.09)	0,2157	0,180396421		20
IMC	0,001	0,89 (0.82-0.96)	-0,2254	0,188508823	(-)	-20
NYHA > 3	1,000	4,2 (2.07-8.34)	0,3886	0,324997909		30

*Probabilidade posterior de que a característica aumente a probabilidade de morte por IC após um ano. Uso do escore: Score = 100 + 1.5age – 15female + 20 leftatrium – 20 BMI + 30 NYHA_3 em que NYHA_3 denota o escore de classificação funcional da New York Heart Association >3. Considerando uma paciente específica, de 45 anos de idade, do sexo feminino, com IC, IMC de 23, e diâmetro do átrio esquerdo de 30, e classificação NYHA =2, o escore de risco de mortalidade um ano pós-alta hospitalar para essa paciente pode ser calculado utilizando-se a seguinte fórmula: Escore=100 + 1,5×45 -15×1 + 20×30 - 20×23 + 30×0=322,5. O escore de risco fica entre a faixa de risco baixo e a de risco moderado.

**Tabela 3:** – Desempenho do modelo nos diferentes escores de risco

Amostra	Modelo baseado em 5 fatores de risco			Modelo baseado em fatores de risco		
	ROC	R-quadrado redimensionado máximo	Teste de Hosmer e Lemeshow	ROC	R-quadrado redimensionado máximo	Teste de Hosmer e Lemeshow
Derivação	0,789	0,1761	0,9013	0,75	0,1159	0,0243
Teste	0,792	0,1514	0,3725	0,771	0,097	0,005
Geral	0,7858	0,1589	0,5945	0,759	0,1069	0,003

### Escore de risco

**Figura 2:**
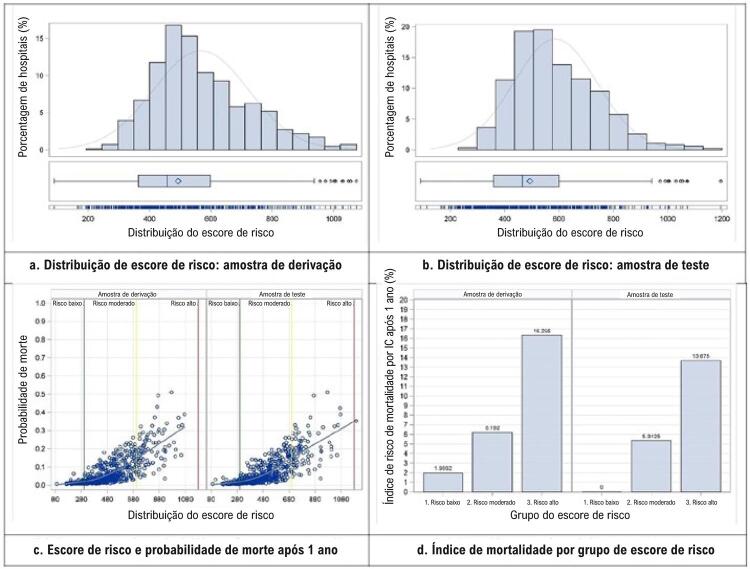
– *Distribuição do escore de risco.*

O escore de risco foi construído utilizando-se as estimativas de CP da Tabela 2 . A equação de cálculo também está listada na Tabela 2 . A média (DP) do escore de risco baseado em amostra de derivação foi de 492,5 (177,1), com uma faixa de 89,9-1195,63, e a média (DP) do escore baseado em amostra de teste foi de 493,0, com uma faixa de 89,9-1073,9. A diferença em escores médios entre as amostras de derivação e de teste não era estatisticamente significativa (p = 0,7324). O escore de risco foi utilizado como variável independente para ajustar a um modelo logístico, o que produziu uma área sob a curva ROC de 0,75 e 0,77 para as amostras de derivação e de teste, respectivamente ( Tabela 3 ). A Figura 2 mostra a distribuição do escore de risco ( Figura 2a e 2b ) e a relação exponencial entre o escore e a probabilidade de mortalidade por IC um ano após a alta hospitalar ( Figura 2c ).

**Figura 3:**
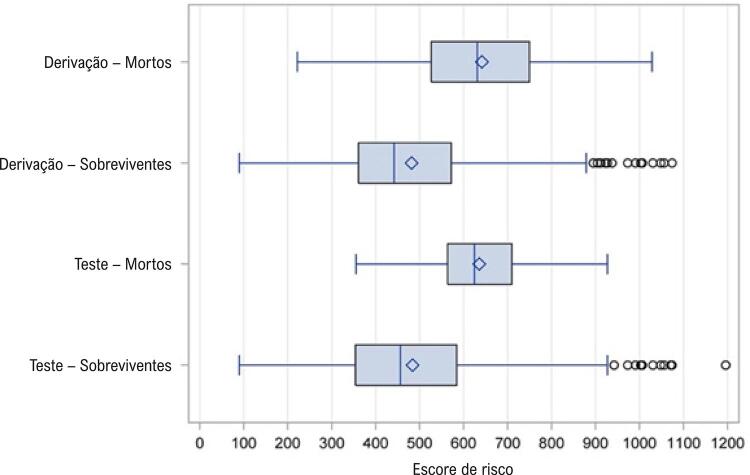
– *Distribuição do escore de risco entre os grupos de óbito e sobreviventes.*

O escore de risco tem boa capacidade preditiva. Com a amostra de derivação, as probabilidades médias previstas para mortalidade após um ano foram de 0,15 e 0,07 para aqueles que morreram dentro do período de um ano e aqueles que sobreviveram após o período de um ano, respectivamente. Esse padrão foi semelhante para a amostra de teste, as probabilidades médias previstas para mortalidade após um ano foram de 0,14 e 0,07 para aqueles que morreram e aqueles que sobreviveram, respectivamente. O escore de risco foi dividido em três faixas baseadas em sua distribuição: (1) risco baixo, se o escore fosse <300; (2) risco moderado se o escore fosse ≥300 e ≤800; (3) risco alto se o escore fosse >800. As proporções de pacientes em cada um desses três grupos de risco foram as seguintes: 11,7% para grupos de risco baixo, 73,8% para grupos de risco moderado, e 14,5% para grupos de alto risco. A Figura 2d mostra as taxas de mortalidade após um ano por risco de grupo e pelas amostras de derivação e de teste. A Figura 3 mostra a distribuição da pontuação de risco por grupos que morreram e sobreviveram.

## Discussão

O desenvolvimento de um modelo de risco de IC específico para a população chinesa é importante, considerando-se que os escores de risco existentes, tais como o amplamente utilizado escore de risco de Framingham, são desenvolvidos principalmente nos Estados Unidos, e não conseguem estimar corretamente o risco em populações asiáticas.^8 - 12^ O presente estudo desenvolveu e validou um escore de risco de 5 variáveis simples para avaliar o risco de mortalidade após um ano em pacientes chineses com IC. Esse escore simples pode ser adicionado à avaliação atual de pacientes antes da alta hospitalar, para proporcionar uma base para que os médicos possam alocar melhor os recursos e identificar pacientes que possam precisar cuidados após a alta. As variáveis finais identificadas no presente estudo foram consistentes com os fatores de risco identificados em coortes ocidentais. Além da idade e da classificação NYHA, que são fatores de risco de mortalidade por IC bem conhecidos, identificou-se que o sexo feminino e o IMC são fatores de proteção dentro do coorte chinês, consistente com os achados baseados na população ocidental.^13 , 14^ Da mesma forma, identificou-se que o diâmetro do átrio esquerdo estava associado à hospitalização por IC e morte.^15^

O número de pacientes dentro do presente coorte foi semelhante aos escores de risco desenvolvidos para outras populações com IC.^16^ Além disso, em comparação com os estudos anteriores, o presente estudo tem vários pontos fortes. Primeiramente, diferentemente de estudos anteriores que avaliaram o risco de mortalidade em curto prazo em pacientes com IC, o presente estudo focou em resultados em longo prazo. Segundo, estes presentes resultados são específicos para pacientes chineses, já que não são derivados de dados obtidos de outros grupos raciais ou étnicos. Portanto, isto se aplica mais a pacientes chineses, em comparação com escores de risco baseados largamente em coortes ocidentais. Por exemplo, o tamanho de efeito entre IMC e mortalidade por IC pode variar entre a população ocidental e a chinesa, uma vez que a população chinesa tem um padrão de gordura corporal diferente do dos grupos populacionais ocidentais. Como resultado, o critério de IMC universal desenvolvidos pela Organização Mundial de Saúde (OMS) não é adequado para a população chinesa e outras populações asiáticas.^17 , 18^ Da mesma forma, o tamanho de efeito entre o diâmetro do átrio esquerdo e a mortalidade por IC entre a população chinesa e a população ocidental pode variar, mostrando que caucasianos geralmente têm um diâmetro do átrio esquerdo maior.^19^ Entretanto, estudos e explorações posteriores são necessários para quantificar essas diferenças. O presente escore foi desenvolvido usando um método estatístico robusto e foi validado utilizando-se dados adicionais. Também houve uma alta concordância com os resultados de derivação. Estas cinco presentes variáveis parecem atender aos critérios de variável ideal. Elas não são afetadas por interpretações clínicas, são amplamente aceitas, estão disponíveis no momento da internação, e podem ser facilmente coletadas.^20^ Por último, ao identificar os componentes modificáveis do presente escore de risco, os serviços de saúde pública podem ser desenvolvidos para resolver esses problemas específicos. Considerando a extraordinariamente grande população da China, esse escore poderia permitir o uso direcionado de recursos médicos e de saúde pública que não inevitavelmente limitados.

O presente estudo tem várias limitações comumente observadas no desenvolvimento de escores de risco baseados em dados clínicos. Isso não leva em conta fatores, tais como a qualidade do médico e do cuidado hospitalar, influências socioeconômicas, ou acesso ao cuidado. Os limiares de risco baixo, intermediário e alto foram baseados naquilo que os pesquisadores consideraram ser risco aceitável dentro de cada categoria. Além disso, informações importantes sobre tratamento, tais como tratamentos farmacológicos e por intervenção, incluindo inibidores de enzima de conversão de angiotensina, bloqueadores de receptores da angiotensina, betabloqueadores, e diuréticos, marca-passos biventriculares, e cardioversores desfibriladores implantáveis, não foram incluídos no modelo. Exames laboratoriais, tais como níveis de hemoglobina e sódio, não foram incluídos no modelo. Finalmente, os pacientes amostrados estavam em 14 hospitais em Pequim, uma das maiores cidades da China. Pode haver diferenças entre as características e o cuidado desses pacientes com IC em relação a residentes de áreas mais rurais ou remotas, que não são levados em consideração. Esse é um achado que foi observado ao se avaliar o risco cardiovascular na China.^21^ Além disso, 42% dos pacientes têm classificação NYHA >3, e o índice de mortalidade é de apenas 6,5%, o que deve ser avaliado em mais detalhes em detalhes futuros. No entanto, embora vários fatores clínicos não tenham sido incorporados à análise, o objetivo do estudo foi desenvolver uma ferramenta simples com variáveis facilmente definíveis para ajudar os médicos.

O presente escore pode ser utilizado para identificar pacientes de alto risco para garantir melhor cuidado após a alta. Dessa forma, ele pode ser usado como guia para médicos planejarem o cuidado de pacientes com IC, derivado de resultados baseados em evidências de pesquisas relevantes e confiáveis. Portanto, os resultados do presente estudo oferecem uma abordagem ao cuidado de saúde, que promove a coleta, a interpretação, e a integração de evidências válidas, importantes e aplicáveis, que estão relacionadas ao paciente, são observadas pelo clínico, e são derivadas de pesquisa, além do uso dessas evidências para ajudar a tomada de decisão.

## Conclusão e direcionamentos futuros

Foi demonstrado um modelo para desenvolvimento de um escore de risco que é diretamente aplicável a pacientes chineses com IC. Isso poderia garantir melhor que fatores genéticos e ambientais de um grupo específico sejam levados em consideração e possam ser usados como estrutura para o desenvolvimento de escores de riscos em outros grupos raciais e étnicos.
