# Supplementary material for: Escore de Risco Clínico Simples para Prever a Mortalidade Pós-Alta Hospitalar em Pacientes Chineses Hospitalizados por Insuficiência Cardíaca
Source: Arq Bras Cardiol. 2021 Aug 2;117(4):615–23. [Article in Portuguese] doi: 10.36660/abc.20200435 (PMC8528360; doi:10.36660/abc.20200435)
Supplement: Translation

# Translation: A Simple Clinical Risk Score to Predict Post-Discharge Mortality in Chinese Patients Hospitalized with Heart Failure

**Authors:** Lei Wang, Li-Qin Wang, Mo-Li Gu, Liang Li, Chen Wang, Yun-Feng Xia

**Affiliations:** 1 China Departamento de Medicina Geriátrica, the Fourth Medical Center, Chinese PLA General Hospital, Beijng - China; 2 China Departamento de Enfermagem, the Eighth Medical Center, Chinese PLA General Hospital, Beijng - China

**Keywords:** Heart Failure, Propensity Score, Mortality, Patient Discharge, Epidemiology

## Abstract

**Background:**

Cardiovascular diseases are the leading causes of death in China. However, present efforts to identify the risk factors for death in patients hospitalized with heart failure (HF) are primarily focused on in-hospital mortality and 30-day mortality in the United States. Thus, a model similar to the model used for predicting the risk in patients considered for cardiovascular surgical procedures is needed to evaluate the risk of the patients admitted with a diagnosis of HF.

**Objective:**

To identify variables that can predict post-discharge one-year HF mortality and develop a risk score to assess the risk of dying within one year.

**Methods:**

In the present study, 1,742 Chinese patients with HF were randomly divided into two groups: a derivation sample group and a test sample group. A Markov Chain Monte Carlo simulation method was used to identify variables that can predict the one-year post-discharge mortality. Variables with a frequency of >1% in the bivariate analysis and that were considered clinically meaningful were eligible for further modeling analyses. The posterior probability that a variable was statistically and significantly associated with the outcome was calculated as the total number of times that the variable’s 95% CI did not overlap with 1 (i.e., the reference point) divided by the total number of iterations. A variable with a probability of 0.9 or higher was considered a robust risk factor for predicting the outcome, and this was included in the final variable list. The level of statistical significance adopted was 5%.

**Results:**

Five variables that could robustly predict the one-year post-discharge mortality were identified: age, female gender, New York Heart Association functional classification score >3, left atrial diameter, and body mass index. Both derivation and test models had a receiver operating curve area of 0.79. These selected variables were used to assess the one-year HF mortality risk score, and these were divided into three groups (low, moderate, and high). The high-risk group corresponds to nearly 86% of the deaths, while the moderate group corresponds to 12% of the deaths.

**Conclusion:**

A simple 5-variable risk score can be used to assess the one-year post-discharge mortality of hospitalized Chinese patients with HF.

## Introduction

Cardiovascular diseases are the leading causes of death in China, accounting for approximately 22.5% of all deaths.^1^ Heart failure (HF) is the twelfth leading cause of hospitalization in China, and four million Chinese suffer from this condition.^1^ Overall, HF in China has an especially poor prognosis, with up to 40% of patients with HF dying within one year.^1^ The financial burden of HF is also substantial.^2^

However, present efforts to identify the risk factors for death in patients hospitalized with HF, such as the Framingham risk score, are primarily focused on in-hospital mortality^3 , 4^ and 30-day mortality in the United States.^5 , 6^ Since HF is a chronic condition, identifying risk factors for the long-term mortality of patients with HF could bring more benefits to patients. A model similar to the model used for predicting the risk in patients considered for cardiovascular surgical procedures can be used to evaluate the risk of the patients admitted with a diagnosis of HF.^7^ Given the increasing burden of HF in China, it is important to find means to stratify patients based on risk, upon initial diagnosis, and upon discharge. Furthermore, with the Asian population encompassing nearly 5% of the population of the United States and the Chinese population representing 20% of the world’s population, the risk scores developed based on Western populations often incorrectly estimate the risk for Asian populations.^8^ Thus, it is important to develop clinically relevant tools for Chinese and other Asian groups. A tool that could specifically provide the likelihood of one-year mortality in Chinese patients with HF would be of great clinical utility because this would have the potential to guide clinical decision-making and identify patients who are more likely to require intensive post-discharge monitoring. Furthermore, given that race, ethnicity, and country of origin have a large impact on clinical outcomes, it is important to develop a risk assessment specific to the group of interest, that is, Chinese patients with HF.

The present study aimed to identify risk factors that are most strongly correlated with one-year mortality among Chinese patients with HF and develop a simple risk score to assess the risk of one-year post-discharge mortality for these patients.

## Methods

### Subjects

The present study has been approved by the Ethics Committee of our hospital, and all patients provided signed written informed consent.

The study cohort was drawn from the Beijing Monitoring Heart Failure Patients and the Building Heart Failure Management Network Study, which included all patients who were ≥20 years of age and hospitalized for HF in one of the 14 designated hospitals in Beijing, China, from October 10, 2015, to October 9, 2017. These patients were randomly assigned into two groups, using a random table method: a derivation sample group and a test sample group. The one-year mortality information was obtained from post-discharge phone interviews.

### Candidate risk variables and outcome variables

The candidate risk variables included the demographic characteristics (age, gender, and body mass index [BMI]), medical history and comorbidities, lifestyle factors, previous cardiac surgeries, clinical findings, and laboratory test results. Age and BMI were measured as continuous variables, while gender was coded as female (yes/no). The medical history included the history of acute myocardial infarction (AMI), history of HF, and history of coronary heart disease (CHD), Type I or Type II diabetes, and hypertension. The previous cardiac surgeries included previous valve surgery. The clinical findings included the New York Heart Association (NYHA) functional classification score (class >3) and left ventricular ejection fraction (<40%). The lifestyle factors included a history of tobacco use, present use of tobacco, and alcohol consumption. The laboratory test data included heart rate, blood pressure, and left atrial diameter (in millimeters), which was measured as a continuous variable.

The outcome variable was the one-year post-discharge HF mortality, which was defined as all-cause deaths that occurred after an index HF hospitalization. The mortality information was obtained by phone interviews with these patients. The last interview date was February 19, 2019. If a patient died within one year after discharge, the date of death was obtained from family members. Patients were excluded from the study sample when neither themselves nor their family members could be reached.

### Statistical analysis

The data of the derivation sample group and test sample group were compared by Chi-square tests for categorical variables and unpaired *t* -tests for continuous variables. Then, clinical judgment and Spearman bivariate correlation analysis were used to identify candidate variables that may be associated with one-year post-discharge mortality. Variables with a frequency of >1% in the bivariate analysis and which were considered clinically meaningful were eligible for further modeling analyses. For observations with missing data, a dummy variable was created to assign a value of 0 when the value of a variable was present, and a value of 1 when the variable was missing. The missing values were then replaced by the median of non-missing values from that continuous variable, and both the dummy and continuous variables were included in the model. This method of modeling the missing data assumed that these data were missing at random, and permitted the inclusion of all available cases, although this was not as efficient as multiple imputation procedures.

The Markov Chain Monte Carlo (MCMC) simulation method was used in conjunction with the logistic regression technique to identify a set of final risk factors for predicting the one-year post-discharge HF mortality. The simulations were carried out with 10,000 iterations for the derivation sample, and a logistic model was fitted for each iteration, yielding a set of variables that are “statistically significant,” or associated with the outcome. Thus, 10,000 iterations of the simulation yielded 10,000 sets of odds ratios (ORs) and 95% confidence intervals (CI), indicating the significance level for each variable’s association with the outcome. The posterior probability that a variable was statistically and significantly associated with the outcome was calculated as the total number of times that the variable’s 95% CI did not overlap with 1 (i.e., the reference point) divided by the total number of iterations. A variable with a probability of 0.9 or higher was considered a robust risk factor for predicting the outcome, and this was included in the final variable list. This method has been used elsewhere.^9^ The area under the receiver operating characteristic (ROC) curve was calculated for each fitted model per iteration to evaluate its discriminating power.^10^

### Development of risk score

Based on the simulation results, a simple risk score was constructed based on the variables selected to assess the one-year mortality. Each variable was weighted using the variable-specific standardized coefficient (SC) obtained from a logistic model based on the original derivation sample, with one-year mortality as the outcome and the selected variables as independent variables. The SC, which measured the change in coefficient for one standard deviation (SD) change in the independent variable, was designed to represent the relative importance of the independent variable in a regression model. This allows for the comparison among independent variables using common units. The risk score for each observation in both derivation and validation samples was calculated as: Score = ∑ Weight_i_⋅ Variable_i_, where weight_i_= SC_i_/ ∑ |SC|, and i = 1, 2, 3, …, total number of the final selected variables. Each weight was then rescaled by 100 to allow the score to be user-friendly in practice, except for the weight for age, which only scales to 10. Each weight was further rounded up or down to its close integer by 5-interval (for example, 32.5 to 30.0, 18.0 to 20.0). Age was rounded down to its close floor with one decimal point. Finally, a base of 100 was added to the score to ensure that there were no negative values in the scores. To validate and test the risk score, two logistic models were fitted. One model used individual variables that were selected from the MCMC simulation as independent variables, while the other model used the risk score as an independent variable. These two models were fitted with both derivation and test samples, and the r-squared and ROC values were calculated from both models to assess the performance of the risk score. All statistical tests were two-sided and had a significance level of 5%, and all analyses were performed using SAS version 9.3 64-bit version (SAS Institute Inc., Cary, North Carolina, USA). All continuous variables were normally distributed as tested by Shapiro–Wilk tests. Continuous variables with normal distribution are described using mean and standard deviation.

## Results

### Patient characteristics

The final study cohort included a total of 1,742 patients with HF. Among these patients, the derivation and test samples included 882 patients and 860 patients, respectively. The mean (SD) age of the cohort was 57.0 (12.5) years, and 9.5% of these patients were 40 or younger, while 30.9% of these patients were 65 or older. Furthermore, 19.9% of these patients were women. The characteristics of patients in the derivation and validation samples were comparable ( Table 1 ). There were no significant differences between these two groups in terms of age, gender, left atrial diameter, heart rate, LVEF <40 (%), AMI (%), NYHA >3 (%), CHD (%), DM (%), HTN (%), valve surgery (%), smoking history (%), alcohol consumption (%), and one-year mortality (%). However, there were significant differences between these two groups in terms of heart rate >100 (%) and HF (%). There was no significant difference in drugs and medical appointments during the follow-up and no significant difference in lab test results among all patients.

**Table 1:** – Baseline characteristics of the study population

Patient characteristics	Overall (n=1742)	Derivation sample (n=882)	Test sample (n=860)	P-value
Demographics Age, mean (SD) years	57.1 (12.4)	57.0 (12.4)	57.1 (12.5)	0.9295
Age missing, n=4 (%)	0.2	0.2	0.2	0.9798
Female (%)	19.9	19.8	19.9	0.9823
BMI, mean (SD)	25.1 (3.4)	25.1 (3.4)	25.0 (3.4)	0.3455
BMI missing, n=0 (%)	0	0	0	
**Clinical findings, and laboratory test**				
Left atrial diameter (mm), mean (SD)	39.9 (7.9)	40.0 (8.1)	39.9 (7.7)	0.823
Left atrial size missing, n=66 (%)	3.8	4.2	3.1	0.1611
Heart rate, mean (SD)	73.0 (14.0)	73.3 (14.8)	72.7 (13.2)	0.8762
Heart rate missing, n=12 (%)	0.7	0.5	0.9	0.2291
Heart rate >100 (%)	4.5	5.6	3.4	0.0276
LVEF <40 (%)	35.5	35.8	35.1	0.7564
LVEF missing, n=0(%)	0	0	0	
**Medical history**				
HF (%)	18.1	20.2	15.8	0.0177
AMI (%)	22.6	23.4	21.9	0.4557
NYHA >3(%)	42.7	42.6	42.8	0.9461
CHD (%)	71.2	70.4	72.0	0.4699
DM (%)	27.8	27.2	28.4	0.5885
HTN (%)	63.6	62.7	64.5	0.4258
Valve surgery (%)	6.1	6.8	5.4	0.2044
**Lifestyle**				
Smoke (%)	57.8	57.6	57.9	0.8956
Alcohol consumption (%)	35.1	34.5	35.8	0.5561
One-year mortality (%)	6.5	7.3	5.8	0.2236

*NYHA: New York Heart Association functional classification score, BMI: Body mass index, CHD: Coronary heart disease, HF: Heart failure, AMI: Acute myocardial infarction, DM: Type I or Type II diabetes, HTN: Hypertension. Continuous data were analyzed by the t-test. Categorical data were analyzed by the chi-squared test. P<0.05 is significant.*

### Risk variables for predicting the one-year HF mortality

The observed one-year mortality rates for the derivation and validation samples were 7.3% and 5.8%, respectively ( *P* = 0.2236). Figure 1 presents the probability that each variable was associated with one-year HF mortality. Five variables, including age, female gender, BMI, left atrial diameter, and NYHA class >3, had a probability of 0.9 or above for significantly being associated with one-year mortality. These were identified as the final variables ( Figures 1a and 1b ). Table 2 illustrates the OR, SC, and 95% CI for each of the five selected variables in the derivation dataset. The area under the ROC curve of this 5-variable-based model was 0.789, with an r-squared of 0.1761 and a goodness-of-fit of 0.9013. The predictive ability ranged from 0.04 in the lowest decile to 0.43 in the highest decile, indicating that the model had good discrimination ( Figure 1c ). This model also performed similarly to the test dataset ( Figure 1d and Table 3 ).

**Figure 1:** – *Variable selection and model development for risk score*

**Table 2:** – Risk factors and corresponding weights in the clinical risk score

Patient characteristics	Posterior probability*	Odds ratio (95% CI)	Standardized coefficient	Initial weight	Direction	Rescaled weight
Age, years	0.969	1.03 (1.01-1.05)	0.1864	0.155891946		1.5
Female	0.02	0.44 (0.20-0.97)	-0.1796	0.150204901	(-)	-15
left atrial diameter (mm)	0.993	1.05 (1.02-1.09)	0.2157	0.180396421		20
BMI	0.001	0.89 (0.82-0.96)	-0.2254	0.188508823	(-)	-20
NYHA > 3	1.000	4.2 (2.07-8.34)	0.3886	0.324997909		30

*Posterior probability that the characteristic increases the probability of one-year HF death. Use of the score: Score = 100 + 1.5age – 15female + 20 leftatrium – 20 BMI + 30 NYHA_3 where NYHA_3 denotes the New York Heart Association functional classification score >3. Giving a particular 45-year-old female HF patient with BMI of 23 and left atrial diameter of 30, and NYHA classification=2, the risk score of one-year post HF discharge mortality for this patient can be calculated as Score=100 + 1.5×45 -15×1 + 20×30 - 20×23 + 30×0=322.5. The risk score is between the low and moderate range.

**Table 3:** – Model performance of the different risk scores.

Sample	5-risk factor-based model			Risk score-based model		
	ROC	Max-rescaled R-Square	Hosmer and Lemeshow Test	ROC	Max-rescaled R-Square	Hosmer and Lemeshow Test
Derivation	0.789	0.1761	0.9013	0.75	0.1159	0.0243
Test	0.792	0.1514	0.3725	0.771	0.097	0.005
Overall	0.7858	0.1589	0.5945	0.759	0.1069	0.003

### Risk score

The risk score was constructed using the SC estimates in Table 2 . The calculation equation is also listed in Table 2 . The mean (SD) of the derivation sample-based risk score was 492.5 (177.1), with a range of 89.9–1195.63, and the mean (SD) of the test sample-based score was 493.0, with a range of 89.9–1,073.9. The difference in mean scores between the derivation and test samples was not statistically significant (p = 0.7324). The risk score was used as the independent variable to fit a logistic model, which yielded an area under the ROC curve of 0.75 and 0.77 for the derivation and test samples, respectively ( Table 3 ). Figure 2 shows the distribution of the risk score ( Figures 2a and 2b ) and the exponential relationship between the score and the probability of one-year post-discharge HF mortality ( Figure 2c ).

**Figure 2:** – *Distribution of risk score.*

The risk score has a good predictive ability. With the derivation sample, the mean predicted probabilities of one-year death were 0.15 and 0.07 for those who died within the one-year window and those who survived after the one-year window, respectively. This pattern was similar for the test sample, in which the mean predicted probabilities of one-year death were 0.14 for those who died and 0.07 for those who survived. The risk score was divided into three ranges based on its distribution: (1) low risk if the score was <300; (2) moderate risk if the score was ≥300 and ≤800; (3) high risk if the score was >800. The proportions of patients in each of these three risk groups were as follows: 11.7% for low-risk groups, 73.8% for moderate-risk groups, and 14.5% for high-risk groups. Figure 2d shows the one-year mortality rates by risk group, and by the derivation and test samples. Figure 3 shows the distribution of risk score by died and survived groups.

**Figure 3:** – *Distribution of risk score by groups of patients who died and who survived.*

## Discussion

Developing a Chinese-population-specific risk model for HF is important, given that the present risk scores, such as the widely used Framingham risk score, are developed predominantly in the United States, which cannot correctly estimate the risk in Asian populations.^8 - 12^ The present study developed and validated a simple 5-variable risk score for assessing the risk of one-year mortality for Chinese patients with HF. This simple score can be added in the present assessment of patients prior to hospital discharge in order to provide a basis for physicians to better allocate resources and identify patients who may need post-discharge care. The final variables identified from the present study were consistent with the risk factors identified in Western cohorts. In addition to age and the NYHA classification, which are well-known risk factors for HF mortality, it was found that female gender and BMI are protective factors within the Chinese cohort, consistent with findings based on the Western population.^13 - 14^ Similarly, it was found that the left atrial diameter was associated with HF hospitalization and death.^15^

The number of patients within the present cohort was similar to risk scores developed for other HF populations.^16^ Furthermore, compared to the previous studies, the present study has several strengths. First, unlike previous studies that evaluated the short-term risk of mortality in HF patients, the present study focused on long-term outcomes. Second, these present results are specific to Chinese patients because these are not derived from data obtained from other racial or ethnic groups. Therefore, this is more applicable to Chinese patients, when compared to risk scores based largely on Western cohorts. For example, the effect size between the BMI and HF mortality could differ between the Western and Chinese population, because the Chinese population has a different pattern of body fat when compared to the Western population groups. As a result, the universal BMI criteria developed by the World Health Organization (WHO) is not suitable for Chinese and other Asian populations.^17 , 18^ Similarly, the effect size between the left atrial diameter and HF mortality between the Chinese population and the Western population could differ, showing that Caucasians usually have a large left atrial diameter.^19^ Nevertheless, further studies and explorations are required to quantify such differences. The present score was developed using a robust statistical method and was validated using additional data. There was also a high agreement with the derivation results. These five variables appear to meet the criteria for an ideal variable. These are unaffected by clinical interpretation, widely accepted, available on admission, and easily collected.^20^ Last, by identifying the modifiable components of the present risk score, public health services can be developed to resolve these specific issues. Given the extraordinarily large population of China, this would allow for the focused use of medical and public health resources that are unavoidably limited.

The present study has several limitations commonly observed in the development of clinic-based risk scores. This does not take into account such factors as the quality of physician and hospital care, socioeconomic influences, or access to care. The thresholds for low-, intermediate-, and high-risk were based on what the investigators considered was an acceptable risk within each category. In addition, important treatment information, such as pharmacological and interventional treatments, including angiotensin-converting enzyme inhibitors, angiotensin receptor blockers, beta-blockers, and diuretics, biventricular pacemakers, and automatic implantable cardioverter-defibrillator devices, were not included in the model. Laboratory evaluations, such as hemoglobin and sodium levels, were also not included in the model. Finally, the patients sampled were from 14 hospitals in Beijing, a major city in China. There may be differences between the characteristics and care of these patients with HF and those residing in more rural or remote areas that are not accounted for, which is a finding that has been observed when assessing cardiovascular risk in China.^21^ In addition, 42% of patients are NYHA >3, and mortality is only 6.5%, which should be further evaluated in future studies. Nevertheless, although a number of clinical factors were not incorporated into the analysis, the purpose of the study was to develop a simple tool with easy-to-define variables to assist physicians.

The present score can be used to identify high-risk patients to provide better post-discharge care. As such, this could be employed as a guide for physicians to plan for the care of patients with HF, derived from evidence-based results of relevant and reliable research. Thus, the results of the present study provide an approach to healthcare, which promotes the collection, interpretation, and integration of valid, important, and applicable patient-reported, clinician-observed, and research-derived evidence, and the use of this evidence for better decision-making.

## Conclusion and future directions

The present study showed a model for developing a risk score that is directly applicable to Chinese patients with HF. This model could better ensure that group-specific genetic and environmental factors are taken into account and can be used as a framework for developing risk scores in other racial and ethnic groups.
